# Research progress in ocular manifestations of hemophagocytic lymphohistiocytosis: Clinical features, mechanisms and therapeutic perspectives (Review)

**DOI:** 10.3892/br.2026.2208

**Published:** 2026-09-30

**Authors:** Weizhen Wu, Yifan Zhou, Ruofei Wu, Ming Yang, Yanling Wang, Kang Wang

**Affiliations:** Department of Ophthalmology, Beijing Friendship Hospital, Capital Medical University, Beijing 100050, P.R. China

**Keywords:** hemophagocytic lymphohistiocytosis, ocular manifestations, uveitis, multimodal imaging, scoping review

## Abstract

Hemophagocytic lymphohistiocytosis (HLH) is a cytokine storm-driven immune dysregulation syndrome in which ocular involvement remains critically underreported. Although isolated case reports have documented ocular findings since 1968, no prior work has systematically mapped the full phenotypic spectrum across all ocular structures, to the best of our knowledge. This gap spans both ophthalmology and hematology, where such manifestations are routinely misattributed to primary infection, autoimmunity or neoplasia. Addressing this lack of information, the present scoping review consolidates 40 peer-reviewed sources via a structured PubMed scoping search, providing the first dedicated synthesis of HLH ocular disease, to the best of our knowledge. Among the published case reports, uveitis, particularly intermediate uveitis or panuveitis, was frequently reported, and retinal pathology encompassed hemorrhage, vascular occlusive changes, serous detachment and distinct acute macular neuroretinopathy-like outer retinal atrophy. Optic nerve involvement may present as disc edema or optic neuritis, occasionally antedating systemic diagnosis, and orbital inflammation frequently mimics cellulitis. Multimodal imaging, including optical coherence tomography, optical coherence tomography angiography and fluorescein fundus angiography, delineates microstructural and microvascular alterations, contextualizing infectious etiologies, such as cytomegalovirus, human herpesvirus 8 and fungal retinitis, alongside primary HLH phenotypes. Consistent with the heterogeneous nature of the current evidence, the present scoping review maps these phenotypes without differentiating between direct HLH-mediated injury and sequelae related to immunosuppression or therapy. Despite the absence of standardized classifications, these findings underscore that ocular assessment may be informative for the early recognition and interdisciplinary management of atypical HLH presentations.

## 1. Introduction

Hemophagocytic lymphohistiocytosis (HLH), also known as hemophagocytic syndrome, is a life-threatening syndrome characterized by uncontrolled, excessive activation of the immune system. Its core pathological mechanism involves the hyperactivation of cytotoxic T cells, natural killer (NK) cells and macrophages, leading to the enhanced release of inflammatory cytokines (such as IFN-γ, TNF-α and IL-6) and the triggering of a ‘cytokine storm’, which may result in multi-organ dysfunction or failure (1).

HLH is categorized into primary [familial, often associated with gene mutations such as in perforin 1 (PRF1) and UNC13D] and secondary (triggered by infections, malignancies and autoimmune diseases) types (2). The diagnosis of HLH primarily relies on the HLH-2004 criteria (3) or the H-Score (4). Treatment focuses on controlling excessive inflammation [such as by using the HLH-94/2004 chemotherapy protocols (3,5)] and addressing the underlying cause (such as via hematopoietic stem cell transplantation) (3,6).

The eye, as an immune active tissue, can also become involved during the course of HLH. Accumulating evidence has indicated that HLH can cause numerous types of ocular damage, including uveitis, retinal hemorrhage and optic neuropathy (7-11). Notably, these observations are predominantly derived from case reports and small case series, rather than systematic epidemiological data. Due to the nonspecific nature of these ocular manifestations and the complex clinical presentation of HLH itself, ophthalmologists often find it challenging to differentiate HLH from infectious, autoimmune or neoplastic eye diseases, leading to misdiagnosis or oversight (8).

Our previous research demonstrated that ocular involvement in HLH is extensive, with vitreoretinal hemorrhage being the most common finding (12). In this previous single-center analysis, factors such as advanced age, autoimmune abnormalities and cytopenia were identified as potential risk factors. These findings suggest that early recognition of ocular lesions may be crucial for raising clinical suspicion of HLH and improving visual prognosis. Therefore, building upon these preliminary observations, the present scoping review aimed to summarize data from 40 studies retrieved from the PubMed database (https://pubmed.ncbi.nlm.nih.gov). The specific aims were as follows: i) To comprehensively characterize the spectrum and clinical features of ocular involvement across different HLH subtypes and patient populations; ii) to explore the underlying pathophysiological mechanisms linking systemic immune dysregulation to site-specific ocular damage, and integrate current diagnostic and therapeutic strategies; and iii) to appraise existing knowledge gaps and propose future research directions to facilitate early recognition, differential diagnosis and timely multidisciplinary management of HLH-related ocular complications.

## 2. Aims and study selection process

### Study objectives

The present scoping review aims to map the phenotypic spectrum of ocular manifestations in patients with HLH. Given the predominantly descriptive nature of the available literature, a scoping review methodology was selected to chart reported phenotypes rather than to evaluate intervention efficacy. The review was framed around three elements: Population, patients with a confirmed diagnosis of HLH (primary or secondary); concept, ocular manifestations involving any ocular structure; and context, clinical practice and published literature without geographical restriction.

### Search strategy and study selection

Although not pre-registered in any public registry (such as PROSPERO), the present review followed a predefined protocol. A comprehensive literature search was performed using PubMed on March 1, 2026, with no restrictions on publication date or language. The search strategy combined the index term ‘Hemophagocytic Lymphohistiocytosis’ with seven ocular-specific key words using the Boolean operator ‘AND’: i) Ocular AND hemophagocytic lymphohistiocytosis (31 records); ii) hemophagocytic lymphohistiocytosis AND eye (33 records); iii) hemophagocytic lymphohistiocytosis AND retina (37 records); iv) hemophagocytic lymphohistiocytosis AND uveitis (19 records); v) hemophagocytic lymphohistiocytosis AND conjunctivitis (7 records); vi) hemophagocytic lymphohistiocytosis AND cornea (3 records); and vii) hemophagocytic lymphohistiocytosis AND eyelid (6 records). A total of 136 records were identified before deduplication. After removal of 36 duplicates, 100 unique records remained for title and abstract screening; 49 were excluded at this stage, leaving 51 reports for full-text assessment. A total of 11 reports were excluded because ocular manifestations were described too briefly or vaguely to permit data charting, leaving 40 sources for inclusion. The study selection process is illustrated in a Preferred Reporting Items for Systematic reviews and Meta-Analyses-style flow diagram adapted for scoping reviews (Fig. 1) (13). Following the removal of duplicate records, two investigators independently screened the titles and abstracts of the remaining articles. Potentially eligible full-text articles were subsequently assessed against predefined eligibility criteria. Discrepancies at any stage were resolved through consensus; when agreement could not be reached, a third senior investigator acted as an arbitrator.

### Eligibility criteria

The present review included peer-reviewed studies reporting on patients with a confirmed diagnosis of HLH (encompassing both primary and secondary forms) who presented with any ocular manifestations. Given the descriptive and heterogeneous nature of the existing evidence, the study deliberately did not differentiate between ocular involvement directly attributed to HLH pathophysiology, and ocular involvement potentially secondary to chemotherapy, hematopoietic stem cell transplantation or opportunistic infections. Articles were excluded if they were: i) Reviews, editorials or non-peer-reviewed reports; ii) cases where HLH was suspected but not definitively diagnosed; iii) studies focusing exclusively on systemic manifestations without detailing ocular findings; or iv) duplicate publications.

### Data charting

Data were extracted using a standardized, piloted form capturing the following information: i) Study characteristics (author, publication year and country); ii) patient demographics (age and sex); iii) HLH characteristics (etiology and diagnostic criteria); iv) clinical context of ocular onset (such as at initial presentation, during systemic therapy and post-transplant); v) specific ocular phenotypes, laterality and ancillary imaging findings; vi) therapeutic interventions; and vii) visual and systemic outcomes. Given the nature of the present scoping review, a formal risk-of-bias assessment was not performed. The characteristics of the 40 included studies are summarized in Table I, with additional details provided in Table SI. Table I presents a summary of key study characteristics, whereas Table SI provides the complete dataset for each study; therefore, some overlap in core items (such as author, year and country) is intentional to allow each table to stand alone.

### Attribution and evidence limitations

The present scoping review deliberately mapped reported phenotypes rather than assigning definitive causal categories. The primary literature consists almost entirely of case reports and small, single-center series, which lack uniform denominators, systematic ophthalmologic screening, and standardized reporting of HLH activity, cytopenias, coagulation status, infectious triggers, transplant history and therapy timelines. Consequently, while definitive causal attribution for individual cases is precluded by these limitations, the present review descriptively distinguishes ocular manifestations directly related to HLH pathophysiology from those arising through other mechanisms, including triggers, systemic complications, immunosuppression, transplantation, treatment effects or coincidental conditions, without attempting independent causal reclassification. Quantitative descriptors (such as ‘frequent’ and ‘common’) reflect patterns in the published literature rather than population-based frequencies. Prognostic assertions reported from individual cohorts are summarized as originally described, with methodological limitations explicitly noted.

## 3. Mapping of reported ocular manifestations in HLH

The following summary is organized anatomically, proceeding from the uvea to the retina, optic nerve, ocular surface and adnexa, followed by a summary of infectious complications. This sequence reflects the relative frequency of reported cases in the literature, moving from sites with comparatively richer phenotypic descriptions (such as the uvea and retina) to less frequently documented but clinically important entities. Notably, the present scoping review aims to map the breadth of reported phenotypes rather than establish pathophysiological hierarchies. Consequently, while a definitive causal classification is precluded by the limitations of the primary literature, the following summary descriptively distinguishes between ocular manifestations presumed to be directly related to HLH and those arising through other mechanisms, such as triggers, systemic complications, immunosuppression, transplantation or treatment effects.

### Uveal involvement

Uveitis is among the most frequently described ocular phenotypes in published case reports and small case series of HLH (12,14,15); however, robust denominators are lacking. Available case reports and series describe involvement of the uveal tract, with histopathological studies identifying inflammatory cell infiltration of the choroid as a prominent feature (7,14).

Pathological observations include a postmortem ophthalmic examination of three adults with HLH, which revealed bilateral uveal tract infiltration by CD163-positive histiocytes with a sparse lymphocytic component; focal hemophagocytosis was identified in certain specimens (15). Inflammatory cells also extended into the trabecular meshwork in one case, prompting the authors to speculate on a potential contribution to aqueous outflow dysfunction and secondary glaucoma (15). These findings provide histopathological evidence that ocular changes observed in HLH can extend beyond the retina to involve anterior segment structures and outflow pathways.

There are extreme hemorrhagic complications reported beyond inflammatory infiltration, with structural compromise of the choroidal vasculature observed. Dsouza and D'souza (16) illustrated a very large suprachoroidal hemorrhage in a patient with dengue-associated HLH, interpreted as a complication potentially associated with thrombocytopenia and vascular permeability under combined viral and hemophagocytic stress; the report described a single case and did not provide multimodal imaging characterization. This case expands the phenotypic spectrum to include catastrophic hemorrhagic events within the uvea, distinct from the serous detachments or cellular infiltrates more commonly described (12).

There is an association between clinical presentation and putative mechanisms: The clinical manifestations of uveitis complicating HLH are heterogeneous and likely multifactorial, with possible differences in involvement patterns between primary and secondary HLH. A 2025 case report and literature review by Alzuabi *et al* (17) described distinct uveal involvement patterns across HLH subtypes. In the reported case of familial HLH with a PRF1 mutation, the patient presented with intermediate uveitis, and fluorescein angiography (FA) revealed perivascular exudation suggestive of choroidal and retinal vessel involvement. Conversely, in infection-related [such as Epstein-Barr virus (EBV)] secondary HLH reviewed by the authors, it was suggested that uveal lesions tend to be more exudative, with severity often coincident with the intensity of the systemic inflammatory response. The authors postulated that these phenotypic differences may reflect distinct underlying mechanisms, although a causal relationship cannot be established from the available data.

Reports further illustrate the diversity and importance of uveal lesions in HLH (18). For example, a 6-year-old child initially presented with bilateral panuveitis, retinal detachment (no light perception in the left eye) and vasculitis in the right eye. Initial treatment with high-dose steroids for uveitis improved inflammation in the right eye; however, subsequent systemic symptoms led to a confirmed diagnosis of primary HLH associated with a RAB27A mutation (19). Moreover, Ducci *et al* (20) reported a case of a 26-year-old man with suspected miliary tuberculosis and coexisting HLH who presented with blurred vision in the left eye; fundus FA raised suspicion of a choroidal tuberculoma. Autopsy reports of familial HLH have also revealed diffuse lymphohistiocytic infiltration in ocular tissues, which can involve the choroid, retina and optic disc, leading to changes such as retinal detachment and optic nerve edema (21). Another phenotype was demonstrated in a case reported by Yao *et al* (22), involving a 51-year-old male patient with HLH. Ocular examination revealed multiple serous pigment epithelial detachments in the left fundus, accompanied by macular edema and scattered punctate hemorrhages. FA showed irregular patchy choroidal fluorescence in the early phase, and multiple punctate leakage and staining at the level of the pigment epithelium in the late phase, consistent with uveitis. The visual acuity of the left eye of the patient recovered well after systemic steroid treatment; however, 9 days after treatment initiation, the contralateral eye developed similar fundus findings. The authors noted that this temporal association raised the possibility of disease progression or, alternatively, that systemic dexamethasone may have contributed to immunosuppression, potentially enhancing replication of latent pathogens [such as cytomegalovirus (CMV)] and thereby contributing to bilateral ocular involvement.

Uveal lesions represent a prominent ocular manifestation in HLH, and appear to differ in pattern between primary and secondary forms. These lesions manifest diversely, ranging from intermediate uveitis and panuveitis to serous pigment epithelial detachment. Therefore, for clinically encountered uveitis of unknown etiology, clinicians should consider HLH in the differential diagnosis, especially when it is bilateral, accompanied by systemic inflammatory signs (such as persistent fever, cytopenia and hepatosplenomegaly) or if there is a poor response to conventional therapy.

### Retinal involvement. Retinal hemorrhage

Retinal hemorrhage has been described in case reports and series regarding HLH, presenting in varied forms including preretinal, intraretinal (flame-shaped or dot blot) and vitreous hemorrhages (23). Postmortem ocular examinations have also documented these varied hemorrhage patterns (18).

A retrospective analysis of 1,525 patients with HLH at a single center identified retinal hemorrhage in 49/341 symptom-directed ophthalmologic examinations (14.4% of examined patients) (12). In this cohort, retinal hemorrhage was markedly associated with decreased red blood cell counts, thrombocytopenia and elevated fibrinogen on multivariate analysis, suggesting an association with HLH-related cytopenias rather than a standalone pathogenic process. Similarly, Tian *et al* (24) reported that retinal hemorrhage was associated with increased mortality risk in their cohort; however, whether it represents an independent prognostic factor or merely a surrogate marker of severe systemic disease remains to be validated.

Isolated case reports have described variable outcomes following ocular interventions. Small amounts of sub-internal limiting membrane hemorrhage have been observed to be self-limiting, with spontaneous resolution reported in select instances (23). However, in cases where hemorrhage persists despite systemic therapy, surgical intervention has been documented. For example, Guilcher *et al* (25) reported a case of EBV-associated HLH where subhyaloid hemorrhage failed to clear despite systemic response to therapy. The patient underwent bilateral vitrectomy, with subsequent improvement in visual acuity. In this case, despite successful visual rehabilitation, the patient ultimately succumbed to complications associated with stem cell transplantation and EBV reactivation. Therefore, based on current descriptive evidence, clinical management should prioritize systemic stabilization over local ocular intervention. Moreover, it has been suggested that addressing underlying systemic risk factors, specifically correcting anemia and coagulopathy (such as stabilizing hemoglobin and platelet levels), is essential prior to considering elective ocular procedures. Analogous observations in severe aplastic anemia suggest that local treatment targeting only the hemorrhage may be insufficient when systemic hypoxia persists (26).

*Retinal vascular occlusion.* Retinal vascular ischemic changes, including appearances resembling central or branch retinal vein/artery occlusion, Purtscher-like retinopathy or peripheral nonperfusion, are among the ocular manifestations described in HLH case reports (12,23). Proposed mechanisms in the literature attribute these complications to cytokine-mediated vascular endothelial damage, a systemic hypercoagulable state and microthrombus formation (27-30).

Observational findings from clinical reports, including those from our previous research, describe fundus presentations resembling central retinal vein occlusion or Purtscher-like retinopathy in patients with HLH (23). Non-invasive imaging via optical coherence tomography angiography (OCTA) has documented hypoperfusion within the retinal capillary and choroidal layers in affected eyes (23). Lin *et al* (10) characterized an adult with HLH who presented with retinal hemorrhages and cotton-wool spots; OCTA revealed foveal avascular zone enlargement, superficial and deep retinal capillary nonperfusion, and patchy choriocapillaris hypoperfusion, thereby corroborating microvascular dropout in both retinal and choroidal circulations in a non-pediatric case (10). Separately, fluorescein fundus angiography (FFA) in HLH cohorts has demonstrated capillary nonperfusion and delayed filling, with Wang *et al* (12) reporting perivascular leakage and disc hyperfluorescence alongside nonperfusion areas. Finn *et al* (31) reported on the case of an infant with HLH and a PRF1 mutation; FFA demonstrated peripheral nonperfusion, vascular dragging and telangiectasia, which was attributed to anemia/coagulopathy and differentiated from discrete occlusion by resemblance to familial exudative vitreoretinopathy/retinopathy of prematurity disorders. Matsubara *et al* (32) described retinopathy with cotton-wool spots in a child with HLH and congenital dysfibrinogenemia, whereas Sebrow *et al* (33) reported spectral-domain (SD)-OCT-confirmed Purtscher retinopathy in an adult with HLH, suggesting that disseminated intravascular coagulation secondary to cytokine storm may contribute to its microvascular pathogenesis. Similarly, Barreiro-González *et al* (34) described Purtscher-like retinopathy with cotton-wool spots and flame hemorrhages in an 11-year-old girl with coexisting dermatomyositis and HLH. Because the patient also met the criteria for an autoimmune connective tissue disease, the extent to which the retinal microangiopathy reflected HLH-mediated cytokine injury, dermatomyositis-associated vasculopathy or their overlap could not be determined; the report is therefore cited here as an illustrative example of Purtscher-like changes in the broader HLH-associated spectrum, rather than as direct evidence of HLH-specific retinal occlusion.

For management observations, published case reports describe heterogeneous ocular interventions, invariably alongside systemic therapy. Anti-VEGF agents (such as intravitreal bevacizumab) have been administered in isolated cases. Notably, in a 2023 report of secondary HLH with retinal vasculitis, combined intravitreal bevacizumab and retinal laser photocoagulation, concurrent with systemic immunosuppression, were followed by preserved visual acuity (20/20) at the 12-month follow-up (35). Moreover, laser photocoagulation has been applied to areas of retinal nonperfusion or neovascularization, as in the Finn *et al* (31) case where panretinal laser was performed for bilateral nonperfusion, with follow-up FFA showing reduced leakage. However, no controlled studies exist, to the best of our knowledge; all descriptions are single case observations during ongoing systemic immunosuppression, and the literature lacks comparative data on outcomes attributable to ocular therapy alone.

*Retinal detachment.* Published case reports have described retinal detachment in patients with HLH, typically in the serous or exudative form. Lubin *et al* (21) reported on two siblings with suspected familial erythrophagocytic lymphohistiocytosis in whom postmortem ocular examination revealed diffuse lymphohistiocytic infiltration of the choroid and retina, associated with retinal detachment and optic disc edema. In a separate clinical report, Lee and Mieler (36) described a 31-year-old woman with hemophagocytic syndrome who developed bilateral blurred vision prior to systemic diagnosis; fundus examination showed multiple serous pigment epithelial detachments and macular edema, with FA demonstrating corresponding leakage. No ocular intervention was pursued due to the critical systemic condition of the patient (36). Furthermore, a 51-year-old man with HLH was reported to exhibit multiple serous pigment epithelial detachments on fundoscopy (22). These reported detachments were often bilateral, ranging from localized subretinal fluid accumulation to extensive serous neurosensory retinal detachment. Narrative reviews have posited that the systemic inflammatory state in HLH, including elevated proinflammatory cytokines, may increase choroidal vascular permeability and disrupt the blood-retinal barrier, thereby favoring subretinal fluid accumulation (27,30); however, direct histopathological confirmation of this sequence in living patients remains lacking.

In a single center retrospective cohort, Wang *et al* (12) identified serous retinal detachment among the spectrum of ocular phenotypes in patients with HLH, often in the context of hypoalbuminemia. In a clinical series employing multimodal imaging, Wu *et al* (23) observed that certain patients with HLH exhibited SD-OCT findings ranging from retinal pigment epithelium (RPE) structural abnormalities and subretinal fluid to serous detachment, morphologically resembling central serous chorioretinopathy (CSC). In individual patients within that series, subretinal fluid was present prior to HLH-specific therapy and partially absorbed following systemic chemotherapy and glucocorticoid treatment. The authors noted this temporal association might aid in differentiating HLH-related detachment from idiopathic CSC.

Literature reports characterize HLH-associated detachment as typically bilateral and extensive, whereas idiopathic CSC is typically unilateral and localized, and is often associated with corticosteroid exposure. In published cases, ocular management has been limited to observation. Lee and Mieler (36) described withholding local intervention due to systemic instability, whereas Wang *et al* (12) and Wu *et al* (23) documented fluid absorption following systemic chemotherapy and glucocorticoids, without performing ocular surgery directed at the serous detachment itself. To the best of our knowledge, no case report in the current HLH literature describes tractional retinal detachment attributable to HLH pathophysiology, and no literature-based recommendation exists for prophylactic local surgery in serous detachment.

*Outer retinal changes*. In a clinical series using multimodal imaging, Wu *et al* (23) observed outer retinal structural alterations in certain patients with HLH, characterized on high-resolution SD-OCT by diffuse or focal ellipsoid zone disruption/thinning, outer retinal atrophy and hyperreflective changes. Near infrared reflectance imaging showed well-defined dark lesions, often darker at the periphery than centrally, morphologically reminiscent of acute macular neuroretinopathy (AMN). The distribution in patients with HLH was not confined to the macula and included the midperipheral retina, with lesions tending to be bilateral and more extensive than typical type 2 AMN (23). The authors postulated that these findings may reflect photoreceptor layer injury secondary to microcirculatory disturbance in the deep retinal capillary plexus or immune-mediated damage during the HLH-associated cytokine storm, mechanisms previously proposed in the general HLH pathophysiology literature (23,29,37). Bhavsar *et al* (38) noted that idiopathic AMN is often associated with putative triggers, such as vasospasm or adrenergic stimulation, situating it within a clinical context distinct from the systemic inflammatory milieu of HLH. No histopathological confirmation of outer retinal infarction in HLH exists to date, to the best of our knowledge.

### Optic nerve involvement

Optic nerve involvement has been reported in a subset of patients with HLH and is often associated with concurrent central nervous system (CNS) disease. A neuroimaging series characterizing CNS-HLH in children documented corresponding optic nerve sheath enhancement, intrinsic optic nerve signal change or secondary optic disc edema in individual cases (39). Pathophysiological mechanisms proposed in the literature include direct inflammatory infiltration of the optic nerve sheath or parenchyma, optic disc edema secondary to elevated intracranial pressure, and microcirculatory compromise driven by the systemic cytokine storm (30,40).

In clinical reports (9,11,39), optic neuropathy in HLH has manifested as optic neuritis or optic disc edema, with the latter appearing more frequently in published descriptions (12). Notably, case reports spanning infants to adults have been published. Petersen and Kuwabara described a 16-month-old infant with familial lymphohistiocytosis exhibiting optic disc edema and vitreous opacities (41). Kawamura *et al* (42) reported a 10-year-old child with known primary HLH who presented with bilateral visual impairment and eyelid swelling; examination revealed bilateral optic disc edema, retinal hemorrhage and perivenous sheathing. Shekarchian *et al* (11) detailed a 9-year-old child with HLH and bilateral optic nerve infiltration on imaging; despite chemotherapy, severe vision loss persisted. Chong *et al* (9) described the case of an 8-month-old child with MRI evidence of optic nerve infiltration during isolated CNS reactivation of HLH. Schafer *et al* (43) reported on the case of a 19-year-old man whose initial presentation was unilateral complete vision loss; imaging revealed optic nerve inflammation initially mimicking idiopathic optic neuritis, with the diagnosis of HLH established only after systemic symptoms emerged. These cases illustrate that unexplained optic neuritis, particularly when accompanied by fever or systemic inflammation, may warrant consideration of HLH in the differential diagnosis.

However, histopathological evidence supporting direct ocular infiltration remains limited to postmortem studies. In an analysis of three adults with HLH and intraocular involvement, Vizcaino *et al* (15) identified focal infiltrates of lymphocytes and histiocytes involving the optic nerve, retina and anterior chamber angle, providing rare histological confirmation of inflammatory invasion in this context.

In summary, published observations characterize HLH-associated optic neuropathy as heterogeneous, ranging from disc edema to optic neuritis, and occasionally serving as the initial presenting feature of atypical HLH. Given its association with underlying CNS pathology, comprehensive assessment, including neuroimaging and cerebrospinal fluid analysis, is typically undertaken when optic nerve symptoms arise. Ophthalmologists should maintain clinical vigilance for HLH in patients presenting with optic nerve inflammation of unclear etiology, integrating ocular findings with systemic status and paraclinical investigations.

### Ocular surface involvement

In a single-center retrospective cohort of 1,525 patients with HLH, 341 underwent ocular examination and 133 had ocular abnormalities; among these, conjunctivitis was recorded in 34 patients (25.56%) and keratitis in 16 (12.03%), while dacryoadenitis and orbital inflammatory lesions were less frequent (12). These figures indicate that ocular surface findings are reported in a minority of examined cases, predominantly in the context of secondary HLH, post-transplant states or concurrent autoimmune disease, rather than as a defining feature of primary HLH.

### Conjunctivitis

Conjunctival involvement has been described as bilateral conjunctival injection in individual HLH reports. Dumke *et al* (44) reported on the case of a 51-year-old man with secondary HLH due to icteric leptospirosis in whom bilateral conjunctivitis was noted alongside fever, jaundice and petechial rash. In autoimmune-associated HLH (such as adult-onset Still's disease and systemic lupus erythematosus), conjunctivitis has been observed to coexist with anterior segment inflammation; post-hematopoietic stem cell transplantation conjunctivitis has also been documented, though often attributable to graft-vs.-host disease or infection rather than HLH itself (12).

*Corneal involvement.* Wang *et al* (12) recorded superficial punctate keratitis in 16 patients with HLH, identifiable on fluorescein staining. Mark and Ma (45) reported on the case of a 26-year-old man with NK/T-cell lymphoma-associated HLH who developed a spontaneous left corneal perforation without preceding trauma or typical infectious infiltration; the authors proposed that histiocyte-mediated tissue destruction, which is analogous to HLH involvement of other ocular tissues, was the most likely mechanism, while acknowledging alternative contributors such as matrix metalloproteinase imbalance or cytokine cytotoxicity. The patient underwent corneal patch grafting, with visual acuity recovering from no light perception to 20/30 at 3 months. This single case illustrates that corneal perforation, although rare, has been described in HLH-associated systemic disease.

*Dry eye disease*. García-Montoya *et al* (46) described the case of a 70-year-old woman with primary Sjögren's syndrome who developed CMV-triggered HLH; the case report notes a history of dry eye attributable to the underlying Sjögren's disease, with no separate quantification of HLH-specific lacrimal dysfunction. Beyond this report, to the best of our knowledge, the published literature does not provide independent epidemiological data separating HLH-related dry eye from that of comorbid autoimmune disease. Moreover, lacrimal gland inflammation (dacryoadenitis) was recorded in 2/341 examined patients with HLH in the study cohort of Wang *et al* (12).

### Eyelid and orbital involvement

Inflammatory involvement of the eyelids and orbits has been documented in HLH, often mimicking infectious cellulitis or idiopathic orbital inflammation. In a retrospective cohort, Wang *et al* (12) recorded orbital inflammatory lesions in 2/341 examined patients and noted eyelid swelling in isolated cases, without attributing these findings to a specific infectious or neoplastic process in all instances. Baba *et al* (47) reported on the case of a 37-year-old man with left periorbital swelling refractory to intravenous antibiotics; histopathological analysis revealed mixed lymphoplasmacytic and histiocytic infiltration, leading to a subsequent diagnosis of HLH, with clinical improvement following immunosuppressive therapy. Laezza *et al* (48) described a patient with unrecognized X-linked lymphoproliferative syndrome type 1 (SH2D1A mutation) who presented with bilateral periorbital swelling resembling orbital cellulitis; the biopsy showed lymphohistiocytic infiltration, and HLH was diagnosed concurrently. Additionally, Giardino *et al* (49) reported EBV-driven incomplete HLH involving the orbits and paranasal sinuses in a patient with XLP1, with a marked response to rituximab. Ramos da Silva *et al* (50) documented sino-nasal-orbital fungal necrosis in a child with HLH, illustrating that fungal invasion can coexist with or mimic inflammatory orbital presentations in this population. Moreover, Lee *et al* (51) reported a case of NK/T-cell lymphoma with secondary HLH presenting as orbital inflammatory disease, suggesting that eyelid and orbital swelling may represent malignancy, infection, or direct inflammatory infiltration, rather than a single etiology.

### Ocular infection complications

Ocular infections have been reported in patients with HLH predominantly in the context of systemic immunosuppression, chemotherapy, hematopoietic stem cell transplantation or the inciting infectious trigger (such as EBV). For the purpose of the present scoping review, these infectious complications are enumerated separately from primary ocular phenotypes (such as uveitis and serous detachment) to address the methodological requirement for separating direct disease manifestations from treatment-related or trigger-related sequelae, as emphasized in contemporary evidence synthesis guidance; however, a definitive distinction between HLH-mediated injury and infection-related injury remains difficult to establish in individual case reports.

Wang *et al* (12) described a patient with EBV-associated HLH who developed CMV retinitis during systemic therapy; aqueous humor PCR was positive for CMV, and fundus examination revealed yellowish white necrotic retinal lesions with hemorrhages. Shekarchian *et al* (11) reported on the case of a 9-year-old child with HLH who presented with neuroretinitis, although a specific causative pathogen was not identified in that report. Valade *et al* (52) reported on the case of an adult with human herpesvirus 8 (HHV8)-associated HLH in whom binocular retinitis-like lesions and anterior chamber flare prompted aqueous PCR confirmation of HHV8, illustrating that ocular findings may flag an atypical viral trigger in secondary HLH. Matsumoto *et al* (53) documented CMV retinitis in an infant following cord blood transplantation, occurring despite negative CMV antigenemia, consistent with reactivation under post-transplant immunosuppression. Akiska *et al* (54) described an adolescent with newly diagnosed HIV/AIDS and disseminated histoplasmosis who developed secondary HLH; while ocular histoplasmosis was not the primary focus, the case underscores the risk of fungal co-infection in immunocompromised patients with HLH. Rathnayake *et al* (55) reported an immunocompetent adult in whom disseminated tuberculosis and secondary HLH coexisted, with fundus examination revealing lesions termed choroidal tubercles that resolved with anti-tuberculosis therapy and corticosteroids. Notably, in the absence of ocular histopathology, it remained unclear whether these lesions represented isolated mycobacterial granulomas, direct HLH-associated histiocytic infiltration, or an overlap of both. Beyond CMV, published case reports have implicated other herpesviruses and *Toxoplasma gondii* in HLH-associated retinitis (11,12). Notably, to the best of our knowledge, the current literature does not provide controlled data to determine whether a given retinal infection arises from the HLH pathophysiology itself or from the associated immunosuppressive state.

### Multimodal imaging modalities. OCT

High-resolution OCT has been utilized to characterize structural alterations in HLH-related retinal lesions. In a retrospective cohort, Wang *et al* (12) documented OCT findings including subretinal fluid (appearing as a hyporeflective space between the neurosensory retina and the RPE), outer retinal atrophy with ellipsoid zone disruption and macular edema manifesting as retinal thickening or cystoid changes. In a subsequent clinical series, Wu *et al* (23) expanded the phenotypic spectrum using SD-OCT, observing that patients with HLH could exhibit a continuum from RPE structural abnormalities to serous detachment, with certain cases morphologically resembling CSC. These structural details provide a basis for monitoring disease progression.

*OCTA*. OCTA has been applied to evaluate the retinal microcirculation in patients with HLH, offering a non-invasive alternative to dye-based angiography. Lin *et al* (10) characterized a case of HLH with retinal hemorrhages and cotton-wool spots, in whom OCTA revealed foveal avascular zone enlargement, capillary non-perfusion in the superficial and deep retinal plexuses, and patchy choriocapillaris hypoperfusion, thereby documenting microvascular dropout in both retinal and choroidal layers. In a larger clinical series, Wu *et al* (23) similarly reported choroidal hypoperfusion (reduced or absent flow signals in the choriocapillaris) in patients with HLH examined with OCTA, and noted that quantitative vessel density metrics could be derived from the built-in software, although such analyses remain descriptive given the case-based evidence. While OCTA facilitates visualization of non-perfusion areas in cases described as retinal vascular occlusion, the literature currently lacks controlled comparisons of OCTA metrics against disease activity scores in HLH.

*FFA*. FFA has been employed to delineate retinal vascular dynamics in HLH. Wang *et al* (12) described FFA findings such as perivascular leakage, optic disc hyperfluorescence and areas of non-perfusion in patients with uveitis or retinal vascular involvement. In specific case reports, FFA has demonstrated vascular dragging and telangiectatic leakage in infants with HLH-associated ischemic retinopathy (39), as well as late-phase leakage corresponding to serous pigment epithelial detachments (36). These angiographic features assist in identifying areas of retinal non-perfusion, although the literature lacks comparative studies validating FFA against clinical outcomes in HLH.

## 4. Discussion

Published case reports and series describe ocular involvement across all segments of the eye in HLH, including the uvea, retina, optic nerve, ocular surface and adnexa. The phenotypic spectrum ranges from anterior segment inflammation (such as conjunctivitis and keratitis) to posterior segment manifestations, such as serous retinal detachment, outer retinal atrophy and optic disc edema. Multimodal imaging, including OCT, OCTA and FFA, has documented corresponding structural and vascular alterations, such as ellipsoid zone disruption, choriocapillaris hypoperfusion and capillary non-perfusion (10,12,22).

Given that ocular involvement in HLH may precede systemic diagnosis or manifest as the initial presenting feature, as demonstrated by cases of optic nerve infiltration antedating hematologic confirmation (9) and uveitis as the first presenting sign (14,19), ocular phenotypes may serve as critical early clues for HLH recognition. Therefore, it may be proposed that patients presenting with unexplained uveitis, retinal vascular alterations or orbital inflammation, particularly when accompanied by constitutional symptoms such as persistent fever, should prompt systematic evaluation for HLH using established diagnostic criteria [for example, HLH-2004 guidelines (3)]. Multimodal imaging, including OCT, OCTA and FFA, may facilitate early detection of subclinical retinal microvascular injury, enabling timely referral for systemic workup. However, it should be acknowledged that the evidentiary basis for these screening strategies derives exclusively from retrospective case reports; prospective multicenter studies are needed to validate the sensitivity and specificity of ocular phenotypes as early markers of HLH, and to establish evidence-based thresholds for triggering systemic HLH evaluation.

Proposed pathophysiological mechanisms in the literature are multifaceted. Narrative reviews posit that the systemic cytokine storm, driven by overactivated T cells and macrophages, may increase vascular permeability and disrupt the blood-ocular barrier, potentially contributing to uveitis and exudative retinal changes (27,30). Concurrent hypercoagulability and endothelial damage have been hypothesized to underlie retinal vascular occlusions and ischemic optic neuropathy (32,33). In secondary HLH, infectious triggers (such as EBV and CMV) or malignancies may directly invade ocular tissues or sustain immune-mediated damage (11,12,45). However, direct histopathological confirmation of these mechanistic links in living patients remains scarce, and no single unifying pathway has been established to explain the heterogeneity of ocular presentations.

Biomarker studies in HLH have focused predominantly on serum markers. Ferritin, IL-18 and glycosylated ferritin have been reported as systemic biomarkers with high specificity for HLH diagnosis in pediatric populations (56). While proteomic profiling of serum is ongoing, analogous analyses of ocular fluids (aqueous humor and vitreous) or tears in patients with HLH are absent from the published literature. Consequently, the potential for intraocular fluid biomarkers to reflect the systemic disease state in real time remains speculative.

Notably, the current body of evidence regarding ocular manifestations in HLH is characterized by methodological constraints. First, the literature consists almost entirely of isolated case reports or small retrospective case series; large-scale, multicenter prospective studies are lacking (12,23). This limits the ability to derive robust epidemiological data regarding incidence, risk factors or natural history. Consequently, quantitative descriptors used herein (for example, ‘frequent’ and ‘common’) reflect patterns within the published literature rather than true population-based frequencies. Furthermore, the reliance on sporadic case reporting means direct HLH-mediated ocular infiltration, complications of cytopenia or coagulopathy, trigger-related ocular disease (such as EBV, tuberculosis and lymphoma), opportunistic infections under immunosuppression, hematopoietic stem cell transplantation-related complications (such as graft-vs.-host disease), treatment toxicity and coincidental findings could not be reliably distinguished. Third, there is no standardized nomenclature or classification system for HLH-related ocular lesions. Terms such as ‘retinal vasculitis’, ‘vascular occlusion’ and ‘optic neuropathy’ are applied variably across reports, hindering cross study comparisons and precluding meta-analysis. Fourth, histopathological data are limited to postmortem examinations in a handful of adult cases, which do not capture the dynamic, *in vivo* disease processes relevant to most clinical presentations (15).

Therapeutic inferences are similarly constrained. Descriptions of ocular interventions (such as anti-VEGF agents and laser photocoagulation) are confined to single case observations wherein systemic immunosuppression was administered concurrently (31,35). No comparative studies exist to isolate the effect of local ocular therapy from the systemic disease response, to the best of our knowledge. Furthermore, the application of novel systemic agents targeting specific cytokines (such as JAK inhibitors and IFN-γ antagonists) has not been systematically evaluated in the context of HLH-related ocular disease. The absence of controlled trials reflects the rarity of the condition and the ethical complexity of randomizing patients with a life-threatening systemic illness to different ocular treatment options. Prognostic assertions derived from individual cohorts should therefore be interpreted cautiously, recognizing these cumulative methodological constraints.

Critically, the extant evidence base does not support the formulation of HLH-specific ocular diagnostic criteria or therapeutic recommendations. All clinical implications noted herein are derived from descriptive case observations and should not be construed as clinical practice guidance pending prospective validation. Additionally, temporal associations between ocular phenotypic progression and systemic HLH disease activity are rarely documented across the included studies, precluding assessment of whether ocular changes reflect real-time systemic inflammatory burden. The geographic and ethnic representativeness of the reported cases is also unassessed, and the phenotypic spectrum summarized herein may not reflect global HLH ocular involvement patterns due to selection bias toward symptomatic cases.

## 5. Conclusion

The present scoping review provides a comprehensive descriptive map of ocular manifestations in HLH, spanning anterior segment inflammation, posterior segment pathologies, optic nerve involvement, adnexal disease and ocular infectious complications. Multimodal imaging modalities, including OCT, OCTA and FFA, offer valuable phenotypic characterization but lack validated diagnostic utility. The current evidence base is exclusively derived from case-level reports, precluding the establishment of standardized diagnostic criteria, pathogenetic hierarchies or evidence-based therapeutic algorithms. Future multicenter prospective registries are warranted to elucidate causal mechanisms, validate early recognition strategies and develop targeted management approaches for HLH-related ocular disease.

## Supplementary Material

Summary of included studies on ocular manifestations in HLH.

## Figures and Tables

**Figure 1:**
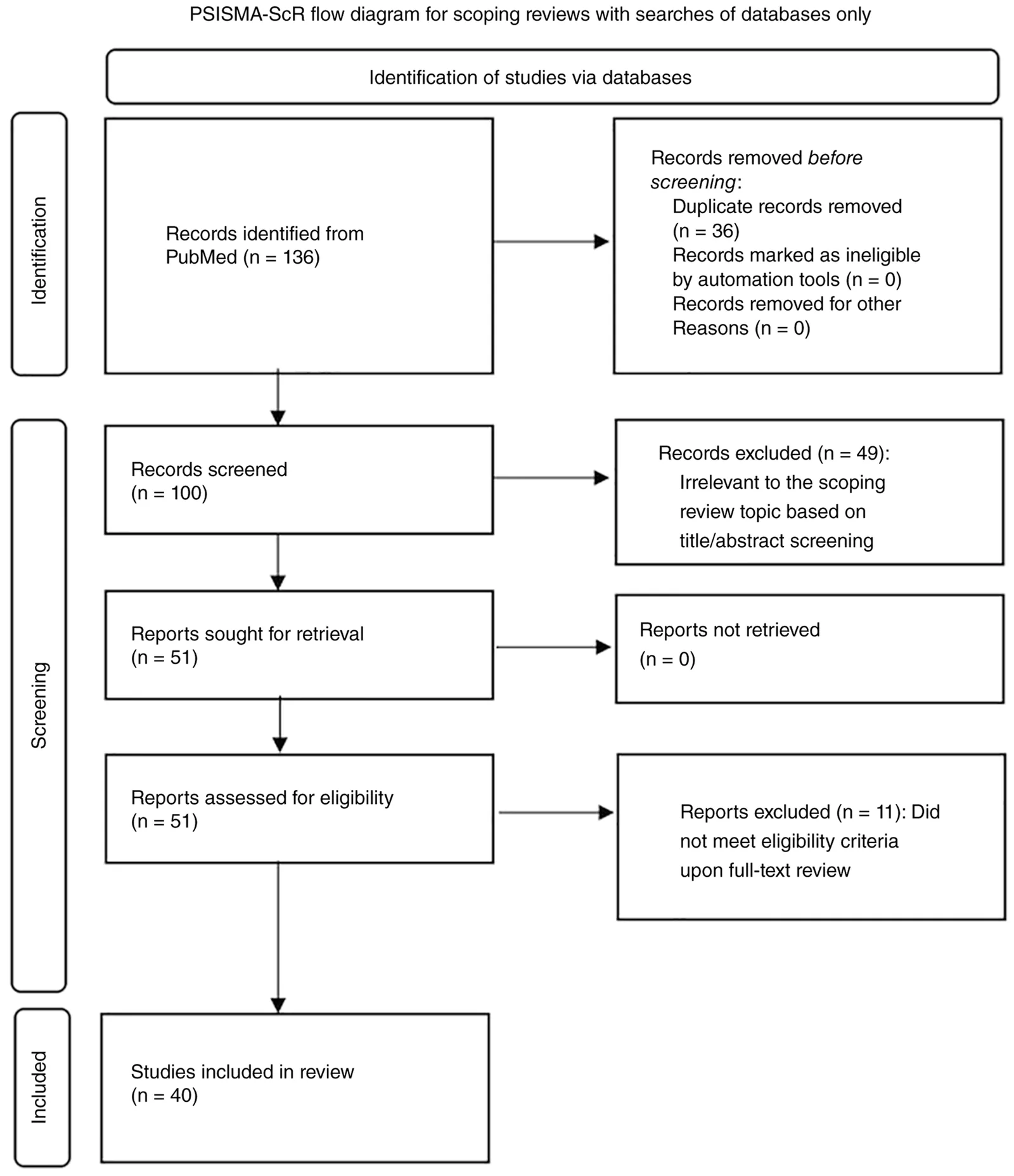
PRISMA-ScR flow diagram of literature selection; adapted from (13). A total of 136 records were identified. After removing duplicates and screening, 51 full-text articles were assessed, of which 11 were excluded due to insufficient detail on ocular findings, leaving 40 sources for inclusion in the present review. PRISMA-ScR, Preferred Reporting Items for Systematic reviews and Meta-Analyses extension for scoping reviews.

**Table I:** Summary of included studies on ocular manifestations in HLH.

No.	First author, year	Country	Study design	No. of patients	Age and sex	Primary/ secondary HLH	Key ocular phenotypes	(Refs.)
1	Park, 1997	USA	Case series	2	Pediatric (19 months and 9 weeks)	Primary	Bilateral optic nerve infiltration, papilledema and choroidal/uveal (trabecular meshwork/iris/ciliary body) infiltration	(7)
2	Suhr, 2016	USA	Case report + LR	1	Pediatric (4 weeks, F)	Primary	Bilateral multifocal deep retinal/RPE/choroidal white dots; partial regression with residual pigmentation at 2-month follow-up	(8)
3	Chong, 2012	Singapore	Case report	1	Pediatric (8 months, F)	Primary	Left optic nerve involvement	(9)
4	Lin, 2019	USA	Case report	1	Adult (30 years, F)	Not specified	Bilateral multiple posterior pole yellow-grey hyperpigmented lesions (<1 DD). OCT: outer retinal atrophy, EZ disruption, choroidal dropout; OCTA: diffuse choriocapillaris hypoperfusion extending beyond lesions, mild hypoperfusion; FFA: early hypofluorescence, late staining; no CNV	(10)
5	Shekarchian, 2023	Iran	Case report	1	Pediatric (9 years, M)	Not specified	Bilateral optic nerve head infiltration/swelling, peripapillary hemorrhage, and epiretinal hemorrhage	(11)
6	Wang, 2023	China	Retrospective case series	341 (with ocular exam; 133 with abnormalities)	Mixed (mean 30.21±14.42 years; median 28 years)	16 primary (4.69%), 325 secondary (95.31%) |	Posterior segment: retinal/vitreous/sub-ILM hemorrhage, serous retinal detachment, CMV retinitis, optic disc swelling, Purtscher-like retinopathy; anterior segment: Uveitis, glaucoma, cataract; adnexal: Orbital cellulitis, pseudotumor, dacryoadenitis, strabismus; ocular surface: Conjunctivitis, keratitis, subconjunctival hemorrhage, chemosis	(12)
7	Li, 2017	China	Case report	1	Adult (53 years, M)	Not specified	Unilateral panuveitis (right eye); keratic precipitates, aqueous flare, vitreous haze, serous pigment epithelial detachment, macular edema)	(14)
8	Vizcaino, 2017	USA	Case series (autopsy)	3	Adult (70, 20 and 49 years, all M)	Not specified	Bilateral uveal infiltration (choroid/ciliary body/iris/trabecular meshwork), with concomitant retinal/optic nerve infiltration, retinal hemorrhages, and focal hemophagocytosis	(15)
9	Dsouza, 2025	India	Case report	1	Adult (44 years, M)	Secondary	Right massive suprachoroidal hemorrhage, conjunctival chemosis, corneal haziness and proptosis	(16)
10	Alzuabi, 2025	Saudi Arabia	Case report + LR	1	Pediatric (11 years, M)	Primary	Bilateral intermediate uveitis, retinal vasculitis, perivascular sheathing, peripheral ‘snowballs’ and acute esotropia	(17)
11	Appen, 1976	USA	Case report	1	Pediatric (5 months, F)	Primary	Bilateral swollen pale optic discs, venous congestion and perivenous yellow-white retinal infiltrates; postmortem H&E: Histiocyte/lymphocyte infiltrate in iris/angle, inner retinal perivascular spaces, prelaminar optic disc and retrolaminar optic nerve; choroid spared	(18)
12	Listernick, 2010	USA	Case report	1	Pediatric (6 years, F)	Primary	Bilateral panuveitis; left retinal detachment; right perivascular exudates and hemorrhages (vasculitis)	(19)
13	Ducci, 2024	Italy	Case report	1	Adult (26 years, M)	Secondary	Left suspected choroidal tuberculoma	(20)
14	Lubin, 1981	USA	Case series	2	Pediatric (7 years, M; 5 years, M)	Primary	Bilateral choroidal effusions, serous retinal detachment, papilledema, anterior uveal infiltration and corneal pannus; postmortem H&E: Choroidal foamy histiocytes, anterior segment (iris/trabecular meshwork) infiltrate	(21)
15	Yao, 2012	China	Case report	1	Adult (51 years, M)	Secondary	Bilateral VKH-like uveitis; serous pigment epithelial detachment, macular edema and scattered retinal hemorrhages	(22)
16	Wu, 2025	China	Retrospective case series	15	Mixed (6-52 years, mean 30.2±10.4 years)	3 primary, 12 secondary	Bilateral/unilateral: Retinal hemorrhage, outer retinal atrophy, ellipsoid zone disruption/thinning, hyperreflective foci, RPE changes and serous retinal detachment	(23)
17	Tian, 2026	China	Retrospective case-control	104	Adult (mean ~37 years, mixed)	3 primary, 101 secondary	Bilateral retinal hemorrhage	(24)
18	Guilcher, 2008	Canada	Case report	1	Pediatric (7 years, F)	Secondary	Bilateral multiple subhyaloid hemorrhages with macular involvement	(25)
19	Finn, 2017	USA	Case report	1	Pediatric (infant)	Primary	Bilateral peripheral retinal nonperfusion, neovascularization, macular/vessel dragging, telangiectasia with late leakage, preretinal tissue contraction, retinal schisis, epiretinal membrane and macular thickening	(31)
20	Matsubara, 1998	Japan	Case report	1	Pediatric (7 years, F)	Secondary	Bilateral vaso-occlusive retinopathy with extensive retinal edema and numerous cotton-wool spots	(32)
21	Sebrow, 2017	USA	Case report	1	Adult (52 years, F)	Secondary	Bilateral Purtscher retinopathy (polygonal inner retinal whitening, flame hemorrhages, periarteriolar sparing)	(33)
22	Barreiro-González, 2018	Spain	Case report	1	Pediatric (11 years, F)	Secondary	Bilateral Purtscher-like retinopathy (cotton-wool spots, venous tortuosity, flame hemorrhages)	(34)
23	Ikić Matijašević, 2023	Croatia	Case report	1	Adult (age NR, M)	Secondary	Bilateral retinal vasculitis, intraretinal hemorrhages, cotton-wool spots, arterial occlusion, capillary non-perfusion, vascular sheathing and left neovascularization	(35)
24	Lee, 2006	USA	Case report	1	Adult (31 years, F)	Not specified	Bilateral VKH-like uveitis; serous pigment epithelial detachment and macular edema	(36)
25	Guandalini, 2014	Australia	Retrospective case series	7	Pediatric (6 weeks- 14 years, 6 M/1 F, mean age 20.8 months in M patients)	Not specified	Bilateral optic nerve thickening/enhancement and retro-orbital soft tissue infiltration, with bilateral retinal detachment	(39)
26	Petersen, 1968	USA	Case report	1	Pediatric (16 months, M)	Primary	Bilateral vitreous haze and fluffy vitreous opacities, papilledema, anterior temporal choroidal white masses, subretinal cellular masses lifting the RPE, inner retinal perivascular infiltrate and iris/ciliary body/trabecular meshwork infiltrate	(41)
27	Kawamura, 1997	Japan	Case report	1	Pediatric (10 years, M)	Not specified	Bilateral optic disk edema, retinal hemorrhages and multiple perivenous white retinal patches	(42)
28	Schafer, 2024	USA	Case report	1	Adult (19 years, M)	Not specified	Right optic nerve infiltration/enhancement (mimicking optic neuritis) with no light perception; left foveal preretinal hemorrhage	(43)
29	Dumke, 2025	Germany	Case report	1	Adult (51 years, M)	Secondary	Bilateral conjunctivitis	(44)
30	Mark, 2025	USA	Case report	1	Adult (26 years, M)	Secondary	Left corneal perforation (inferonasal limbus, 3 mm) with iris prolapse; bilateral severe eyelid edema and chemosis	(45)
31	García-Montoya, 2017	Spain	Case report	1	Adult (70 years, F)	Secondary	Ocular dryness (Sjögren's syndrome)	(46)
32	Baba, 2024	UK	Case report	1	Adult (37 years, M)	Secondary	Left periorbital swelling, conjunctival injection, mild proptosis and left upper eyelid/orbital inflammation (preseptal progressing to orbital)	(47)
33	Laezza, 2025	Italy	Case report + LR	1	Pediatric (5 years, M)	Primary	Bilateral lower eyelid edema/hyperemia and soft mass (right eye more prominent than left), mimicking preseptal orbital cellulitis	(48)
34	Giardino, 2024	Italy	Case report	1	Pediatric (4.5 years, M)	Primary	Bilateral lower eyelid edema/erythema, preseptal orbital cellulitis-like inflammation and right periorbital subcutaneous infiltrate	(49)
35	Ramos da Silva, 2025	Brazil	Case report	1	Pediatric (1 month, M)	Secondary	Bilateral upper eyelid edema progressing to medial orbital, nasal and palatal necrosis	(50)
36	Lee, 2025	Australia	Case report	1	Adult (60s, M)	Secondary	Right periorbital swelling, chemosis, proptosis, ophthalmoplegia and light perception-only vision	(51)
37	Valade, 2019	France	Case report	1	Adult (33 years, F, HIV-positive)	Secondary	Unilateral conjunctival Kaposi's sarcoma	(52)
38	Matsumoto, 2015	Japan	Case report	1	Infant (5 months, M)	Primary	Bilateral multiple white retinal spots (CMV retinitis)	(53)
39	Akiska, 2024	USA	Case report	1	Early adolescent (M)	Secondary	Bilateral optic disc drusen, retinal micro-ischemic changes and cotton-wool spots	(54)
40	Rathnayake, 2015	Sri Lanka	Case report	1	Adult (40 years, F)	Secondary	Bilateral choroidal tubercles; FFA: Late-phase staining	(55)

LR, literature review; HLH, hemophagocytic lymphohistiocytosis; F, female; M, male; DD, disc diameter; NR, not reported; CMV, cytomegalovirus; OCT, optical coherence tomography; OCTA, OCT angiography; FFA, fundus fluorescein angiography; CNV, choroidal neovascularization; RPE, retinal pigment epithelium; EZ, ellipsoid zone; ILM, internal limiting membrane; VKH, Vogt-Koyanagi-Harada disease.

## Data Availability

Not applicable.
