# Supplementary material for: Research progress in ocular manifestations of hemophagocytic lymphohistiocytosis: Clinical features, mechanisms and therapeutic perspectives (Review)
Source: Biomed Rep. 2026 Sep 30;25(5):135. doi: 10.3892/br.2026.2208 (PMC13688156; doi:10.3892/br.2026.2208)
Supplement: Supplementary file 1 [file Supplementary_Data.pdf]

Table SI. Summary of included studies on ocular manifestations in HLH.

No.	First author, year	Country	Study design	No. of patients	Age and sex	Primary/secondary HLH	Underlying etiology	HLH diagnostic criteria	Ocular phenotype	Laterality	Imaging findings	Rx	Visual outcome	Systemic outcome	Major limitations	(Refs.)
1	Park, 1997	USA	Case series	2	Pediatric (19 months and 9 weeks)	Primary	Familial erythrophagocytic lymphohistiocytosis	Clinical/histological (pre-2004)	ON and choroidal infiltration (case 1: Prominent ON; case 2: Prominent choroidal + trabecular meshwork/iris/ciliary body)	Bilateral	Gross pathology + H&E + IHC (MAC387/CD3/CD4) on enucleated eyes; no antemortem imaging	Etoposide + steroids + IT MTX (case 1); supportive only (case 2)	Not assessed (infants)	Died (both)	Small series; no antemortem imaging	(7)
2	Suhr, 2016	USA	Case report + LR	1	Pediatric (4 weeks, F)	Primary	Familial HLH (clinically presumed)	HLH-2004 criteria	Bilateral retinal white dots (deep retina/RPE/choroid)	Bilateral	RetCam: Multiple small white dots throughout macula and beyond vascular arcades; ONs normal; at 2-month follow-up: Increased pigmentation, incomplete resolution	Cyclosporine A + dexamethasone + etoposide; empiric antibiotics; stem cell transplantation	Unknown (died post-transplant)	Died	Single case; no OCT	(8)
3	Chong, 2012	Singapore	Case report	1	Pediatric (8 months, F)	Primary	Genetic: PRF1 mutation	Clinical HLH-2004	ON involvement	OS	MRI: thickened and enhancing OS ON	Dexamethasone + etoposide + CSA → MUD PBSCT	Not assessed	Short follow-up	Single case; MRI only; vision not assessed	(9)
4	Lin, 2019	USA	Case report	1	Adult (30 years, F)	Not specified	Not specified	HLH-2004	Multiple posterior pole lesions <1 DD (yellow/grey hyperpigmented)	Bilateral	Optos imaging: Hyperpigmented lesions; SD-OCT: Outer retinal atrophy, EZ disruption, choroidal dropout; OCTA: Mild SCP/ICP/DCP hypoperfusion, diffuse choriocapillaris hypoperfusion extending beyond lesion; FAF: hypoattenuation; FA: Early hypofluorescence, late staining; no CNV	Dexamethasone + etoposide	Blurry vision OD (stable)	Short follow-up	Single case; no tissue confirmation	(10)
5	Shekarchian, 2023	Iran	Case report	1	Pediatric (9 years, M)	Not specified	Not specified	Prior oncology-confirmed HLH	Bilateral ONH infiltration/swelling, peripapillary hemorrhage, epiretinal hemorrhage	Bilateral	MRI: Bilateral ON + brain tumoral infiltration	Etoposide-based chemotherapy	Vision deteriorated	Short follow-up	Single case; no biopsy	(11)
6	Wang, 2023	China	Retrospective case series	341 (with ocular exam; 133 with abnorm	Mixed (mean 30.21±14.42 years; median	16 primary (4.69%), 325 secondary (95.31%) |	Secondary: infection 64.52%, lymphoma 15.25%, autoimmune 5.87%, other/mixed 9.09%	HLH-2004	Retinal/vitreous hemorrhage, 49; serous RD, 6; CMV retinitis, 1; optic disc swelling, 10; anterior: uveitis, 11; glaucoma, 5; cataract, 1; adnexa:	49 retinal/vitreous hemorrhages: 31 bilateral, 18	Fundus/OCT/OCTA/FA/B-scan/UWF: Sub-ILM hemorrhage, EZ disruption, choriocapillaris hypoperfusion, PAMM/AMN, Purtscher-	HLH protocol (dexamethasone + etoposide ± CSA); ocular Rx supportive/observation	Not assessed per-eye (cohort); some self-limited	Alive/remission not separately reported for ocular subgroup	Retrospective; not all examined; mixed etiologies	(12)

				alities)	28 years)				Orbital cellulitis, 2; pseudotumor, 2; dacryoadenitis, 2; strabismus, 2; surface: Conjunctivitis, 34; keratitis, 16; subconjunctival hemorrhages, 11; chemosis, 5	unilateral; 6 SRD: 5 bilateral; others mixed	like, serous RD with choroidal thickening					
7	Li, 2017	China	Case report	1	Adult (53 years, M)	Not specified	Not specified	Not specified	Unilateral panuveitis OD (KP, flare, vitreous haze, serous PED, macular edema)	OD	Slit lamp: KP 1+, flare 3+, vitreous 2+; OCT: Macular thickness 959 µm, serous PED, macular edema	Oral prednisone (ocular); then IVIG + methylprednisolone + meropenem + supportive	20/200 OD (not reassessed post-systemic collapse)	Died	Single case; HLH unconfirmed	(14)
8	Vizcaino, 2017	USA	Case series (autopsy)	3	Adult (70, 20 and 49 years, all M)	Not specified	Case 1, AIDS/VZV/toxo; case 2, EBV; case 3, none identified	Cases 2 and 3 met ≥5/8 HLH-2004; case 1: met 4/8 HLH-2004 criteria (authors' classification)	All three cases: Bilateral uveal (choroid/ciliary/iris/trabecular meshwork) histiocytic infiltrate; focal extension to retina and ON in some cases. Case 1: Subtle H&E, ciliary body involvement on CD163; case 2: Choroidal and retinal infiltrate; case 3: Focal hemophagocytosis.	Bilateral	Postmortem H&E + CD163/CD3/CD20/BRAF-V600E	None ocular-specific; systemic dexamethasone + etoposide/CSA (case 3)	Not specified	All died	Post-mortem only; no antemortem exam	(15)
9	Dsouza, 2025	India	Case report	1	Adult (44 years, M)	Secondary	Dengue fever	HLH-2004	Massive suprachoroidal hemorrhage; conjunctival chemosis, corneal haziness, proptosis	OD	Hemorrhagic choroid	IV steroids + platelet transfusions; day 9 OD surgical evisceration	OD: NLP (lost); OS: 6/6	Alive	Single case; dengue confounding factor; enucleation	(16)
10	Alzuabi, 2025	Saudi Arabia	Case report + LR	1	Pediatric (11 years, M)	Primary	PRF1 gene mutation	HLH-2004	Intermediate uveitis, retinal vasculitis, perivascular sheathing, peripheral 'snowballs', acute esotropia	Bilateral	UWFFA: Focal vascular leakage, no ischemia; OCT macula normal	IV methylprednisolone + IVIG + plasma exchange → HLH protocol (dexamethasone + etoposide + IVIG) → HSCT transfer	20/20 both eyes	Transferred for HSCT	Single case; concurrent therapies; transferred	(17)
11	Appen, 1976	USA	Case report	1	Pediatric (5 months, F)	Primary	Familial lymphohistiocytosis	Pre-2004 clinical + histopathological	Swollen pale discs, venous congestion, perivenous yellow-white retinal infiltrates; iris/angle	Bilateral	Antemortem fundus only; PM H&E: histiocyte/lymphocyte infiltrate iris/angle/inner retina/prelaminar	IV antibiotics + supportive	Not assessed	Died	Pre-2004; no IHC; vision not assessed	(18)

									histiocytes + erythrophagocytosis, inner retinal perivascular infiltrate, marked ON infiltration, choroid spared		disk/retrolaminar ON					
12	Listernick, 2010	USA	Case report	1	Pediatric (6 years, F)	Primary	RAB27A homozygous mutation	HLH-2004	OS panuveitis, RD (NLP), OD perivascular exudates/hemorrhages (vasculitis) on steroid taper	Bilateral	Retinoblastoma	Dexamethasone + cyclosporine + etoposide + IT MTX + alemtuzumab	OS: NLP (irretrievable); OD: 20/15 initially, later vasculitis (vision not reassessed post-Rx)	Alive	Single case; concurrent steroid taper	(19)
13	Ducci, 2024	Italy	Case report	1	Adult (26 years, M)	Secondary	Miliary tuberculosis	HLH-2004	Suspected choroidal tuberculoma OS	OS	Fundus + FA: Suspicion of choroidal tuberculoma OS	Anti-tuberculosis therapy + dexamethasone	Not recorded	Recovered	Single case; no biopsy; tuberculosis vs. HLH uncertain	(20)
14	Lubin, 1981	USA	Case series	2	Pediatric (7 years, M; 5 years, M)	Primary	Familial erythrophagocytic lymphohistiocytosis	Pre-2004 clinical + histopathological	Bilateral choroidal effusion + serous RD, papilledema (PM), anterior uvea infiltrate (iris/trabecular), corneal pannus; case 1 only	Bilateral	Antemortem: Fundus (choroidal effusions, RD, papilledema); PM H&E: Choroid foamy histiocytes, anterior segment infiltrate	Erythromycin + ADH + steroids (ineffective)	Case1: HM → 20/200 OD, 20/50 OS then died; case 2: Not recorded	Both died	Pre-2004; no OCT	(21)
15	Yao, 2012	China	Case report	1	Adult (51 years, M)	Secondary	Virus-associated (CMV)	HLH-2004	OS serous PED + macular edema + scattered hemorrhages; OD later same pattern; VKH-like uveitis	Bilateral	FA: Early irregular patchy choroidal fluorescence, pinpoint leakage, late staining at RPE	Systemic dexamethasone (ocular first) → ganciclovir + ofloxacin + prednisone	OS: 20/200 → 20/40; OD: 20/20 → 20/50	Died	Single case; CMV vs. HLH vs. VKH uncertain	(22)
16	Wu, 2025	China	Retrospective case series	15	Mixed (6-52 years, mean 30.2±10.4 years)	3 primary, 12 secondary	EBV, 8; familial, HLH; 3; EBV + T/NK lymphoma. 2; EBV + T-cell lymphoma, 1; DLBCL, 1	HLH-2004	Retinal hemorrhage (26 eyes), outer retinal atrophy (20 eyes), EZ disruption (17 eyes), hyperreflective foci (7 eyes), EZ thinning (10 eyes), RPE changes (4 eyes), serous detachment (3 eyes)	Bilateral (13), unilateral (2)	Fundus + Optos imaging: Hemorrhages (superficial/Roth/sub-ILM/flame); SD-OCT: Outer retinal atrophy/EZ loss/thinning/RPE changes; NIR: hypereffective lesions; OCTA (2 eyes): SCP/DCP/choriocapillaris hypoperfusion; FFA/AF (2 eyes):	HLH-94/2004 (dexamethasone + etoposide ± CSA); 2 no systemic Rx at baseline; 3 post-HSCT; no standalone ocular Rx	Not recorded	Not recorded	Retrospective; limited imaging; mixed etiologies	(23)

											Limited; B-scan: Limited					
17	Tian, 2026	China	Retrospective case-control	104	Adult (mean ~37 years, mixed)	3 primary, 101 secondary	Secondary: EBV/infection/malignancy/autoimmune disease	HLH-2004	Retinal hemorrhage	Bilateral	Optos imaging: Punctate/scattered hemorrhages; SD-OCT: Increased CRT/IRT, decreased CVI	HLH-94/2004 protocol (dexamethasone + etoposide ± CSA)	Not assessed	Not recorded	Retrospective; selection bias	(24)
18	Guilcher, 2008	Canada	Case report	1	Pediatric (7 years, F)	Secondary	EBV (PCR+)	HLH-2004	Multiple subhyaloid hemorrhages (macular involvement)	Bilateral	Clinical fundus: Hemorrhages	Pars plana vitrectomy + HLH chemotherapy	Re-vitrectomy 20/400 OD, 20/200 OS → post-vitrectomy 20/20 OD, 20/25+ OS	Died	Single case; hemorrhage mechanism unclear	(25)
19	Finn, 2017	USA	Case report	1	Pediatric (infant)	Primary	PRF1 mutation	HLH-2004	Peripheral retinal nonperfusion, neovascularization, macular/vessel dragging (OD), telangiectasia with late leakage, preretinal tissue contraction, retinal schisis, ERM, macular thickening	Bilateral	Handheld SD-OCT (Bioptigen): OD preretinal elevation/schisis/ERM/thickening; OS normal fovea; FA: OD inferotemporal dragging + nonperfusion + loops, OS peripheral nonperfusion + NV + late leak; color fundus: OD macular drag, OS normal	HLH protocol (pre-BMT) + bilateral panretinal laser photocoagulation (2 sessions)	Fix-and-follow both eyes at 3 months post-laser (no numeric VA)	Alive	Single case; handheld imaging only; short follow-up	(31)
20	Matsubara, 1998	Japan	Case report	1	Pediatric (7 years, F)	Secondary	EBV/CMV/VZV serology only	Henter 1991 diagnostic guidelines + registered HLH-94	Bilateral vaso-occlusive retinopathy: Extensive retinal edema, numerous CWS	Bilateral	Bilateral ophthalmoscopy: Retinal edema + CWS, vessels normal; post-therapy edema resolved; visual field: Central + upper nasal defect	Dexamethasone + etoposide + cyclosporin A	OD 0.08, OS 0.10 (severely reduced, persistent)	Alive	Single case; dual viral trigger; no OCTA	(32)
21	Sebrow, 2017	USA	Case report	1	Adult (52 years, F)	Secondary	EBV + CMV co-infection	HLH-2004	Purtscher retinopathy (polygonal inner retinal whitening, flame hemorrhages, periarteriolar sparing)	Bilateral	Color fundus: Polygonal posterior pole whitening + flame hemorrhages, macular arteriole sparing; SD-OCT: Inner retinal thickening (IPL/INL/OPL), small ONL cystic edema, 1 pocket subretinal fluid OS, 'saddle sign'	IV methylprednisolone + etoposide + rituximab	HM both eyes	Died	Single case; leukostasis vs. infiltrate uncertain	(33)

22	Barreiro-González, 2018	Spain	Case report	1	Pediatric (11 years, F)	Secondary	Dermatomyositis + HLH	Not itemized	Purtscher-like retinopathy (CWS, venous tortuosity, flame hemorrhages)	Bilateral	Fundus: Cotton-wool exudates, venous tortuosity, flame hemorrhages	Systemic corticosteroids	20/20 both eyes, preserved; resolved in 3 weeks no sequelae (later posterior subcapsular cataract from steroids)	Alive, short follow-up	Single case	(34)
23	Ikić Matijašević, 2023	Croatia	Case report	1	Adult (age NR, M)	Secondary	SLE flare + MAS	HLH-2004	Retinal vasculitis, intraretinal hemorrhages, CWS, arterial occlusion, capillary non-perfusion, vascular sheathing, NV (OS)	Bilateral	Fundus: Hemorrhages + CWS; FA: OD temporal non-perfusion/arterial occlusion, OS NV + leak	MP → prednisone + HCQ + MMF → intravitreal bevacizumab (OS) + panretinal laser both + rituximab 1 g x 2	20/20 both eyes (stable at 12 months)	Alive	Single case; SLE/MAS/HLH triplet	(35)
24	Lee, 2006	USA	Case report	1	Adult (31 years, F)	Not specified	Not specified	Not specified	Bilateral serous PED + macular edema (VKH-like)	Bilateral	Color fundus: Posterior pole + nasal serous PED; FA: Pinpoint leakage + late RPE staining; SD-OCT: Macular thickness 845 µm OD, 439 µm OS	None	20/60 OD, 20/40 OS (unchanged at 2-week recheck)	Died	Single case; no HLH subtype	(36)
25	Guandalini, 2014	Australia	Retrospective case series	7	Pediatric (6 weeks-14 years, 6 M/1 F, mean age 20.8 months in M patients)	Not specified	Not specified	Not specified	ON thickening/enhancement (2/7), retro-orbital soft tissue infiltration + bilateral RD (1/7)	Bilateral	Orbit MRI: 2 ONs thickened + enhancing, 1 retro-orbital infiltrate + bilateral RD	HLH protocol (steroids + etoposide + CSA ± ATG), BMT in subset	Not assessed	Not reported per-case	Small series; no pathology	(39)
26	Petersen, 1968	USA	Case report	1	Pediatric (16 months, M)	Primary	Familial lymphohistiocytosis	Pre-2004 clinical + histopathological	Bilateral vitreous haze + fluffy vitreous opacities, bilateral papilledema, anterior temporal choroidal white masses (OS), subretinal cell masses lifting RPE, inner retinal perivascular infiltrate, iris/ciliary/trabecular infiltrate	Bilateral	Postmortem H&E: Vitreous/retina/choroid/ON head/subretinal space lymphocyte + histiocyte infiltrate with hemophagocytosis (lymphocytes/erythrocytes/debris), inner retinal perivascular, RPE lifted	Antibiotics + steroids	Not assessed	Died	Pre-2004; no IHC	(41)
27	Kawamura,	Japan	Case	1	Pediatric (10	Not specified	Not specified	Clinical (pre-	Bilateral optic disk edema, retinal	Bilateral	Fundus: Disk edema + hemorrhages +	IT methotrexate + etoposide-based	OD 20/130 →	Alive	Pre-2004; no OCT/FA;	(42)

	1997		report		years, M)			2004)	hemorrhages, multiple perivenous white retinal patches		perivenous white patches; MRI: Bilateral ON enlargement	chemo → BMT	20/20; OS 6/200 → 20/25		attribution uncertain	
28	Schafer, 2024	USA	Case report	1	Adult (19 years, M)	Not specified	Not specified	HLH-2004	R ON infiltration/enhancement (mimicking optic neuritis), OD NLP; OS foveal preretinal hemorrhage	Bilateral	MRI orbit: OD ON + orbital fat enhancement; OCT RNFL: OD 39 µm (atrophy), OS 109 µm; Humphrey visual field test: OD generalized depression	IVIG + solumedrol + etoposide + dexamethasone PO + IT methotrexate	OD: NLP (stable); OS: 20/25 → preretinal hemorrhage improved	Alive	Single case; no biopsy; thrombocytopenia concurrent	(43)
29	Dumke, 2025	Germany	Case report	1	Adult (51 years, M)	Secondary	Leptospirosis	HScore + HLH-2004	Bilateral conjunctivitis	Bilateral	Not recorded	Tazobactam → ceftriaxone + dexamethasone 10 mg/m^2/d + IVIG 40 g x 3 days + noradrenaline + NIV + hemodialysis	NR	Recovery	Single case; conjunctivitis nonspecific	(44)
30	Mark, 2025	USA	Case report	1	Adult (26 years, M)	Secondary	Extranodal NK/T-cell lymphoma + EBV	HLH-2004	OS corneal perforation (inferonasal limbus, 3 mm, iris prolapse), no infiltrate; bilateral severe eyelid edema/chemosis; OD eye normal	OS	External photo: Necrotic facial mass, periorbital edema, OS corneal perforation + iris incarceration; intraoperative: 3-mm limbal perforation, iris prolapse, clear remainder cornea	Corneal tectonic patch graft (donor tissue) + subconjunctival dexamethasone/cefazolin + topical steroids/antibiotics	OS: 20/200 → 20/100 (1 week) → 20/30 (3 months); OD: 20/200 (limited by edema, later normal)	Alive	Single case; perforation mechanism unclear	(45)
31	García-Montoya, 2017	Spain	Case report	1	Adult (70 years, F)	Secondary	Primary SS + acute CMV infection	HLH-2004	Ocular dryness (SS)	Not recorded	None	IV ganciclovir → methylprednisolone 500 mg x 3 days → cyclosporine 6 mg/kg + hydroxychloroquine 200 mg BID + artificial tears	Not recorded	Recovered	Single case; SS vs. HLH vs. CMV	(46)
32	Baba, 2024	UK	Case report	1	Adult (37 years, M)	Secondary	Intra-abdominal sepsis (VRE/ESBL 4 years prior) + pancreatitis + reactive HLH	HLH-2004	OS periorbital swelling, conjunctival injection, mild proptosis, OS upper eyelid/orbital inflammation (preseptal → orbital)	OS	CT orbit: OS periorbital swelling to lateral globe, no collection/sinus disease; MRI orbit: Similar; biopsy (lid fat/lacrimal/orbicularis): lymphoplasmacytic + histiocytes, $CD3^+$/$CD79a^+$/$CD68^+$/sc	IV meropenem + teicoplanin + tigecycline → anakinra 2 mg/kg BID → dexamethasone PO + anakinra 200 mg BID	6/4.5 R, 6/7.5 OS (stable throughout)	Recovered	Single case; sepsis confounded; concurrent Rx	(47)

											attered CD30$^+$/MCT$^+$					
33	Laezza, 2025	Italy	Case report + LR	1	Pediatric (5 years, M)	Primary	XLP1	HLH-2004	Bilateral lower eyelid edema/hyperemia, soft mass (OD>OS), mimicking preseptal orbital cellulitis	Bilateral	MRI orbit: T2 hyperintense thickening periorbital subcutaneous fat, ethmoid-maxillary sinus obliteration; biopsy: CD2/CD3/CD5/CD8$^+$ T cells, CD68$^+$ histiocytes, EBER$^+$	Dexamethasone + anakinra + acyclovir → rituximab → HSCT	Stable (vision preserved)	Alive	Single case; sinusitis complicates; no fundus	(48)
34	Giardino, 2024	Italy	Case report	1	Pediatric (4.5 years, M)	Primary	XLP1	HLH-94/2004	Bilateral lower eyelid edema/redness, preseptal orbital cellulitis-like, OD periorbital subcutaneous infiltrate	Bilateral	MRI orbit/brain: Ethmoid-maxillary sinus obliteration, periorbital subcutaneous thickening T2-hypo (OD>OS); eyelid biopsy: CD3/CD8 T-cells + CD68 histiocytes, EBER$^+$, Ki67 ~70%, no CD30/CD1a/S100	Dexamethasone 10 mg/m^2 + anakinra + acyclovir → rituximab (EBV titer drop) → HSCT	Not assessed	Alive	Single case; EBER+ vs. HLH uncertain	(49)
35	Ramos da Silva, 2025	Brazil	Case report	1	Pediatric (1 month, M)	Secondary	Neonatal bacterial infection + invasive Aspergillus fumigatus	HLH-2004	Bilateral upper eyelid edema → medial orbital/nasal/palate necrosis	Bilateral	MRI orbit: Contrast loss midline, sino-orbital necrosis; biopsy: Septate hyphae (*Aspergillus*)	Dexamethasone 2 mg/day + etoposide 2.5 mg/kg + ruxolitinib 4 mg/day + liposomal amphotericin B 10 mg/kg	Not assessed	Died	Single case; fungal, not HLH-direct	(50)
36	Lee, 2025	Australia	Case report	1	Adult (60s, M)	Secondary	Extranodal NK/T-cell lymphoma	2009 modified HLH criteria + HScore	OD periorbital swelling, chemosis, proptosis, ophthalmoplegia, OD LP only	OD	CT: OD medial/inferior rectus fusiform enlargement + proptosis; MRI: Persistent muscle enlargement; biopsy: CD2/CD3/Granzyme B/Perforin/TIA-1/CD56$^+$, CD30 20%, PD-L1 60%, MIB-1 80%, EBER$^+$	Dexamethasone + cisplatin + gemcitabine + pegaspargase	OD: 6/6 → LP	Died	Single case; lymphoma invasion, not HLH	(51)
37	Valade, 2019	France	Case report	1	Adult (33 years, F, HIV-positive)	Secondary	HHV8 viremia >8 log + HIV+ + KS conjunctival + plasmablastic B-cell phenotype	HLH-2004	Conjunctival KS	Unilateral	Clinical photo: Conjunctival KS lesion	Etoposide + liposomal doxorubicin (KS)	Not recorded	Alive	Single case; KS is HIV-driven, not HLH	(52)
38	Matsumoto, 2015	Japan	Case report	1	Infant (5 months, M)	Primary	Familial HLH	Not recorded	Bilateral multiple white retinal spots	Bilateral	Anterior chamber PCR+ CMV retinitis; fundus: Bilateral multiple white spots	Ganciclovir (systemic) + dexamethasone	Not assessed, lesions improved	Alive	Single case; CMV coexisted; concurrent Rx	(53)

39	Akiska, 2024	USA	Case report	1	Early adolescent (M)	Secondary	Newly diagnosed HIV/AIDS + histoplasmosis	HLH-2004/HScore	Optic disc drusen, bilateral retinal micro-ischemic changes, CWS	Bilateral	Fundus exam: Optic disc drusen, bilateral micro-ischemic retinal lesions, CWS	Anakinra, ART, liposomal amphotericin B plus itraconazole, and ceftriaxone	Not recorded	Alive	Single case; HIV/infection vs. HLH uncertain	(54)
40	Rathnayake, 2015	Sri Lanka	Case report	1	Adult (40 years, F)	Secondary	Disseminated tuberculosis	HLH criteria	Choroidal tubercles	Bilateral	Fundus photo: Yellowish fleshy choroid tubercles; FA mentioned in report	Anti-tuberculosis (category-1, 9 months) + dexamethasone 2 weeks (initial IV methylprednisolone x 3 days)	Not recorded	Recovered, no relapse at 18 months	Single case; tuberculoma, not HLH infiltrate	(55)

ADH, antidiuretic hormone; AF, autofluorescence; AIDS, acquired immunodeficiency syndrome; AMN, acute macular neuroretinopathy; ART, antiretroviral therapy; ATG, antithymocyte globulin; BMT, bone marrow transplantation; BRAF-V600E, B-Raf proto-oncogene, V600E mutant protein; CNV, choroidal neovascularization; CRT, central retinal thickness; CSA, cyclosporine A; CVI, choroidal vascularity index; CWS, cotton-wool spots; DCP, deep capillary plexus; DD, disc diameter; DLBCL, diffuse large B-cell lymphoma; EBER, EBV-encoded small RNA; EBV, Epstein-Barr virus; ERM, epiretinal membrane; ESBL, extended-spectrum β-lactamase; EZ, ellipsoid zone; F, female; FA, fluorescein angiography; FAF, fundus autofluorescence; FFA, fundus fluorescein angiography; H&E, hematoxylin and eosin; HCQ, hydroxychloroquine; HHV8, human herpesvirus 8; HIV, human immunodeficiency virus; HLH, hemophagocytic lymphohistiocytosis; HM, hand motion; HSCT, hematopoietic stem cell transplantation; ICP, intermediate capillary plexus; IHC, immunohistochemistry; ILM, internal limiting membrane; INL, inner nuclear layer; IPL, inner plexiform layer; IRT, inner retinal thickness; IT, intrathecal; IV, intravenous; IVIG, intravenous immunoglobulin; KP, keratic precipitates; KS, Kaposi sarcoma; LP, light perception; LR, literature review; M, male; MCT, mast cell tryptase; MAS, macrophage activation syndrome; MMF, mycophenolate mofetil; MP, methylprednisolone pulse; MTX, methotrexate; MUD, matched unrelated donor; NIR, near-infrared; NIV, non-invasive ventilation; NK, natural killer; NLP, no light perception; NV, neovascularization; OCT, optical coherence tomography; OCTA, optical coherence tomography angiography; OD, right eye; ON, optic nerve; OPL, outer plexiform layer; OS, left eye; PAMM, paracentral acute middle maculopathy; PBSCT, peripheral blood stem cell transplantation; PED, pigment epithelial detachment; PM, postmortem; PRF1, perforin 1; RD, retinal detachment; RNFL, retinal nerve fiber layer; RPE, retinal pigment epithelium; Rx, treatment; SD-OCT, spectral-domain optical coherence tomography; SCP, superficial capillary plexus; SLE, systemic lupus erythematosus; SRD, serous retinal detachment; SS, Sjögren's syndrome; toxo, toxoplasmosis; UWF, ultra-widefield; UWFFA, ultra-widefield fluorescein angiography; VRE, vancomycin-resistant *Enterococcus*; VKH, Vogt-Koyanagi-Harada disease; VZV, varicella-zoster virus; XLP1, X-linked lymphoproliferative syndrome type 1.
